# Targeting health-related quality of life issues not found with functional outcome measures a year after aneurysm subarachnoid hemorrhage in a Swedish national cohort

**DOI:** 10.1007/s00701-026-07059-z

**Published:** 2026-10-16

**Authors:** Helena Aineskog, Bryndís Baldvinsdóttir, Ola G. Nilsson, Asgeir S. Jakola, Erik Kronvall, Elisabeth Ronne Engström, Paula Klurfan, Peter Alpkvist, Mikael Svensson, Jan Hillman, Peter Lindvall

**Affiliations:** 1 Department of Clinical Sciences - Neurosciences, Umeå University, 901 87 Umeå, Sweden; 2 Department of Clinical Sciences - Neurosurgery, Lund University, Lund, Sweden; 3 Institute of Neuroscience and Physiology, Department of Clinical Neuroscience, Sahlgrenska Academy, University of Gothenburg, Gothenburg, Sweden; 4 Department of Medical Sciences, Section of Neurosurgery, Uppsala University, Uppsala, Sweden; 5 Department of Clinical Neuroscience, Karolinska Institute, Stockholm, Sweden; 6 Department of Clinical Sciences, Linköping University, Linköping, Sweden

**Keywords:** aSAH, EQ-5D-3L, GOSE, HRQoL, National

## Abstract

**Purpose:**

Outcome after aneurysmal subarachnoid hemorrhage (aSAH) is often devastating with life time issues also for independent survivors. We aimed to identify health related quality of life (HRQoL) issues in this favorable functional outcome group compared to a sex and age matched general population.

**Methods:**

We have analyzed data from 634 Swedish aSAH patients from a national prospective study ranging between 2014 and 2018. One year after ictus, functional outcome was analyzed using Glasgow Outcome Scale Extended (GOSE) and HRQoL assessed by Euro-quality of life—5 dimensions—3 levels (EQ-5D-3L). Favorable GOSE (5–8) were found in 471 patients. Age- and sex-matched populations were created by published data from a cross-sectional cohort of 32,597 people living in Stockholm County in 2006. Odds ratios (ORs) for rating a specific EQ-5D-3L level were calculated for each domain. Significance was set at α = 0.001.

**Results:**

Patients reported less mobility problems than the matched general population (9.6% vs 14.7%; OR 0.62 *p* = 0.002). However, with usual activities significantly more difficulties with moderate, (*24.0% vs 10.9%;* OR 2.58, *p* < .001) and any problems (27.0% vs 12.5%; OR 2.58, *p* < .001) were reported. More issues with extreme anxiety/depression were also reported ((5.3% vs 2.8%; OR 1.94; *p* = 0.002). Unfavorable GOSE patients were doing worse than the matched population in all levels and dimensions measured (Online Resource 1, 2, 3 and 4).

**Conclusion:**

Although patients with favorable functional outcome report good or better mobility compared to their age and sex matched general population, they have significantly more issues with usual activities a year after aSAH more extreme issues with anxiety/depression were also reported To our knowledge, this is the first time EQ-5D-3L data is compared in a national group of aSAH patients with favorable functional outcome to a sex and age matched general population.

**Supplementary Information:**

The online version contains supplementary material available at https://doi.org/10.1007/s00701-026-07059-z.

## Introduction

Aneurysmal subarachnoid hemorrhage (aSAH) is a life trauma for many patients and improving health-related quality of life (HRQoL) for survivors remains a central goal [14, 26].

During the last decades, a substantial improvement in survival [29] and improved functional outcome after aSAH has been noted [36]. Although understudied [4], there are concerns as to what extent the growing “favorable outcome” group of patients are doing well or not. Specific symptoms of subarachnoid-stroke survivors have been suggested [40] as fatigue [12], depression and anxiety [14], reduced cognitive function [3], and reduced quality of life [3].

Traditionally, outcome after aSAH is evaluated using functional outcome [41], usually dichotomized as favorable (independent) versus unfavorable based on the need for assistance in daily activities and care. Still many independent patients are not well enough to proceed with a functional private and working life [14, 30, 40]. EQ-5D-3L is a generic HRQoL instrument that is easy to comprehend and perform [7] even for patients with fatigue and cognitive issues and has previously been used to evaluate outcome after subarachnoid hemorrhage [14, 27, 34, 35, 48–50].

For the aSAH group as a whole, all 5 dimensions of EQ-5D are affected a year after aSAH [35]. Ratings improve in all dimensions during the first year after aSAH [27, 49] and then, usual activities continue to improve, whereas the other dimensions remain stationary [49]. All dimensions except pain/discomfort are still affected 10 years after ictus[48].

EQ-5D-3L requires information about the present state of a person independent of circumstances. Age and sex distributions do have substantial effects on HRQoL in all societies [8, 9, 43]. To be disease-specific, therefore, the outcome of EQ-5D-3L must be viewed in the context of the baseline HRQoL in a matched “general population.” This is of specific importance for the aSAH population, as it has an overrepresentation of females and patients are of higher average age than most general populations [56].

To date, few publications have used age- and sex-matched general population comparisons when evaluating EQ-5D-3L outcome after aSAH [27, 48, 49] and no previous study has made such comparisons among independent patients.

In this study, we aim to evaluate if EQ-5D-3L provide any additional information over functional outcome divided into favorable and unfavorable outcome after aSAH compared to a sex and age matched general population. Our hypothesis is that this might reveal areas of interest to find and help independent patients after aSAH to a better life.

## Methods

### Study design and population

This prospective cross-sectional study is based in a national multicenter study involving all neurosurgical departments treating aneurysms in Sweden (10 million inhabitants) between September 2014 and March 2018 (3.5 years). All patients with spontaneous SAH (sSAH) were eligible for inclusion. No information was acquired regarding patients who were not admitted to a neurosurgical department. Ethical approval was granted by the Swedish Ethical Review Authority on February 4, 2014 (1E-99E/EIOZ). Data is not made publicly available as no ethical approval has been granted for data release.

All procedures were performed in accordance with the 1964 Declaration of Helsinki and its later amendments.

Informed consent was obtained from all included patients or an appropriate next of kin. The “Strengthening the reporting of observational studies in epidemiology”** (**STROBE) guideline has been implemented.

Data on one thousand thirty-seven (1,037) patients, aged 18 years or older, with aneurysmal SAH were extracted for this study.

**Fig. 1:**
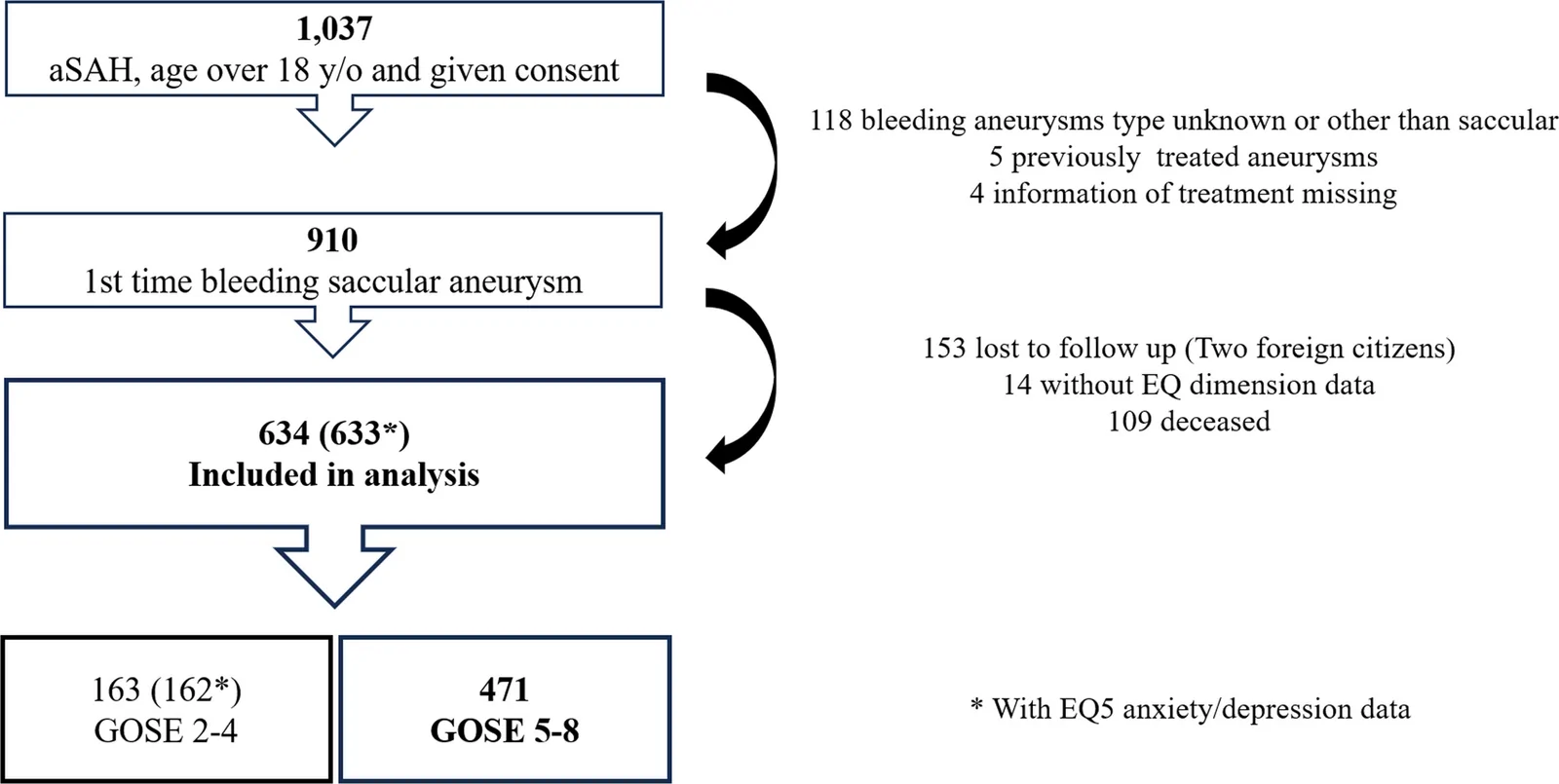
Flowchart of included and excluded patients for EQ-5D-3L analysis. aSAH = aneurysm subarachnoid hemorrhage. y = years old

Exclusion criteria were applied as follows: bleeding from a previously treated aneurysm (5), unknown type or non-saccular aneurysm (118), information on choice of treatment missing (4). Of the 910 patients with 1 st rupture, saccular aneurysm, 153 were lost to follow-up, of whom two were foreign citizens. Yet another 14 patients did not fill out the EQ-5D-3L questionnaire and 109 were deceased at the time of follow-up. The remaining 634 were analyzed in this study, except in dimension 5, anxiety/depression of EQ-5D-3L, where one patient did not answer the question, see Fig. 1.

Patient demographics and clinical characteristics are presented in Table 1 (patients with favorable GOSE outcomes) and Online Resource 1 (patients with unfavorable outcomes and the complete aSAH cohort). Collected baseline data include sex, median age, and age distribution, categorized into an initial 18–24 group followed by 10-year intervals up to 74 years, and a final category for 75 years and older. Premorbid variables encompass smoking status, body mass index (BMI), hypertension, diabetes, previous stroke, psychiatric diagnose, and kidney disease. Upon admission to the neurosurgical department, clinical severity was assessed using the World Federation of Neurological Surgeons (WFNS) scale [33], and the presence of hemiparesis was recorded. The SAH diagnosis was confirmed through a computed tomography (CT) scan or through cerebrospinal fluid analysis. Aneurysm evaluation was performed with a CT angiography and/or digital subtraction angiography (DSA).

**Table 1:** Descriptive characteristics

		Total*	Favorable GOSE *n* = 471	
		*n*		
Age (yrs) median (min–max), IQR		471	56 (19–85)	16
18–24 (yrs) *n*, %		471	7	1.5%
25–34 (yrs) *n*, %		471	21	4.5%
35–44 (yrs) *n*, %		471	57	12.1%
45–54 (yrs) *n*, %		471	138	29.3%
55–64 (yrs) *n*, %		471	131	27.8%
65–74 (yrs) *n*, %		471	94	20%
75 + (yrs) *n*, %		471	23	4.9%
Sex (female) *n*, %		471	342	72.6%
Current smoking, *n*, %		455	149	32.7%
BMI (kg/m^2^) mean (min–max), SD		438	26.0 (16–49)	5.1
Hypertension		470	161	34.3%
Diabetes		470	10	2.1%
Previous stroke		470	12	2.6%
Psychiatric diagnosis		470	36	7.7%
Kidney disease		470	5	1.1%
dWFNS (poor = 3–5)		414	90	21.7%
WFNS grade				
	1		206	49.8%
	2		118	28.5%
	3		8	1.9%
	4		68	16.4%
	5		14	3.4%
dFisher (4)		471	173	36.7%
Hemiparesis		410	20	4.9%
Location		471		
	ICA +		140	29.7%
	MCA		91	19.3%
	Anterior complex		187	39.7%
	Posterior circulation		53	11.3%
Treatment		471		
	Microsurgical		150	31.8%
	Endovascular		319	67.7%
	Both		2	0.4%
Meningitis		468	27	5.8%
DIND		465	83	17.8%
Ictus to treatment (h) median (IQR)		445	80	147
Hospital stay (d) median (IQR)		452	14	9
Admission to follow-up (d) median (IQR)		446	445	206
Seizure		468	19	4.1%
Shunt dependency		461	63	13.7%

Descriptive characteristics of the favorable GOSE (5–8) aSAH population. *Number analyzed in the group. *IQR* interquartile range, *yrs* years, *n* number, *BMI* body mass index, *SD* standard deviation, *d* dichotomized, *ICA* + internal carotid artery and posterior communicating artery, *MCA* middle cerebral artery, *aSAH* aneurysm subarachnoid hemorrhage, *dFisher* dichotomized Fisher grade, *WFNS* world federation of Neurosurgical Societies functional outcome scale, *DIND* delayed ischemic neurologic deficit, *GOSE* Glasgow outcome scale extended

The initial CT scan was reviewed regarding blood distribution using the Fisher scale [18]. Poor dichotomized Fisher grade (dFisher) was defined as grade 4.

Aneurysm location was sorted into one of the four groups as previously described by Jaja et al. [24, 28]. The bleeding source was treated by endovascular intervention, microsurgery or a combination of the two, as assigned independently at each neurosurgical unit.

In addition to treatment modality, confirmed meningitis and delayed ischemic neurological deficit (DIND) during the hospital stay were recorded. Time-dependent variables included ictus-to-treatment time (hours), total hospital stay (days), and the interval from admission to follow-up (days). Finally, late sequelae, such as seizures and shunt dependency, were assessed at follow-up.

### Follow-up

Outcome was measured one year after ictus.

Functional outcome was evaluated based on GOSE [53, 54], dichotomized as unfavorable (dGOSE grade 2–4) or favorable (grade 5–8).

HRQoL was evaluated using EQ-5D-3L. A standardized Swedish questionnaire was used in a face-to-face manner or as a telephone interviewer script. It includes a visual analogue scale, EQ-VAS, where the patient rates the overall perceived HRQoL on a scale from 0 to 100, and the EQ-5D-3L descriptive system [43]. The descriptive system evaluates quality of life in five domains: *Mobility (M), Self-care (SC), Usual activities (UA), Pain/Discomfort (PD),* and *Anxiety/Depression (AD)*.

Each domain is rated as level 1, *“No problems,”* level 2, *“Moderate problems,”* or level 3, *“Extreme problems.*” The collapse of level 2 and 3 is named “*Any problems*.”

### General population data

General population data were retrieved from published data by Szende et al. [43] unchanged as originally described by Sun et al. in 2012 [42]. The original general population included 32,597 people from the age of 18–84 years, representing a cross section of the general population living in Stockholm County and replying to the survey in 2006 (response rate 61%).

The sex- and age-matched general populations were derived through indirect standardization, adopting the same percentage distribution of people as in the full, the favorable and the unfavorable aSAH populations based on sex and age groups of 18–24 years, 25–34 years, 35–44 years, 45–54 years, 55–64 years, 65–74 years, and 75 years and older (75 +). EQ-5D-3L VAS outcomes were not normalized due to lack of publicly available data to generate the normalization model.

### Statistical analysis

IBM SPSS Statistics software (v 26) was used for analyses. Cross-tabulation and Pearson’s chi-square test were used to compare variables between groups. The Mann–Whitney U test was performed to compare distributions between groups as regards median values. The independent t-test was used to compare means, with equal variances assumed and 2-tailed significance applied.

To find a potential knowledge gap between the commonly used dGOSE and EQ-5D-3L data, we compared odds for the favorable and unfavorable GOSE groups separately of rating a certain dichotomization level of EQ-5D-3L for each domain to the odds for their respective matched population. This was done by logistic regression and the differences in odds between the groups were expressed as odds ratios (ORs). Through this procedure, the domains affected by the aSAH in the two groups could be described.

To pinpoint the different interpretations of outcomes between an unmatched, country-, sex-, and age-dependent aSAH group, and the outcome after adjustment for these factors by comparison of odds with those of a sex- and age-matched population, the hierarchical order of OR outcomes was compared with the order of ratings in the aSAH groups without adjustment.

*p* was set at 0.001 (significance level α = 0.001), based on the number of analyses (95) performed.

## Results

### Descriptive data for the populations

The median age among the favorable GOSE patients was 56 years and 72.6% were female. Endovascular treatment was used in: 67.7%. The comorbidities, poor dFisher grade, presence of hemiparesis, need for hematoma evacuation, meningitis, delayed ischemic neurological deficit, seizures, and shunt dependency are detailed in Table 1.

**Fig. 2:**
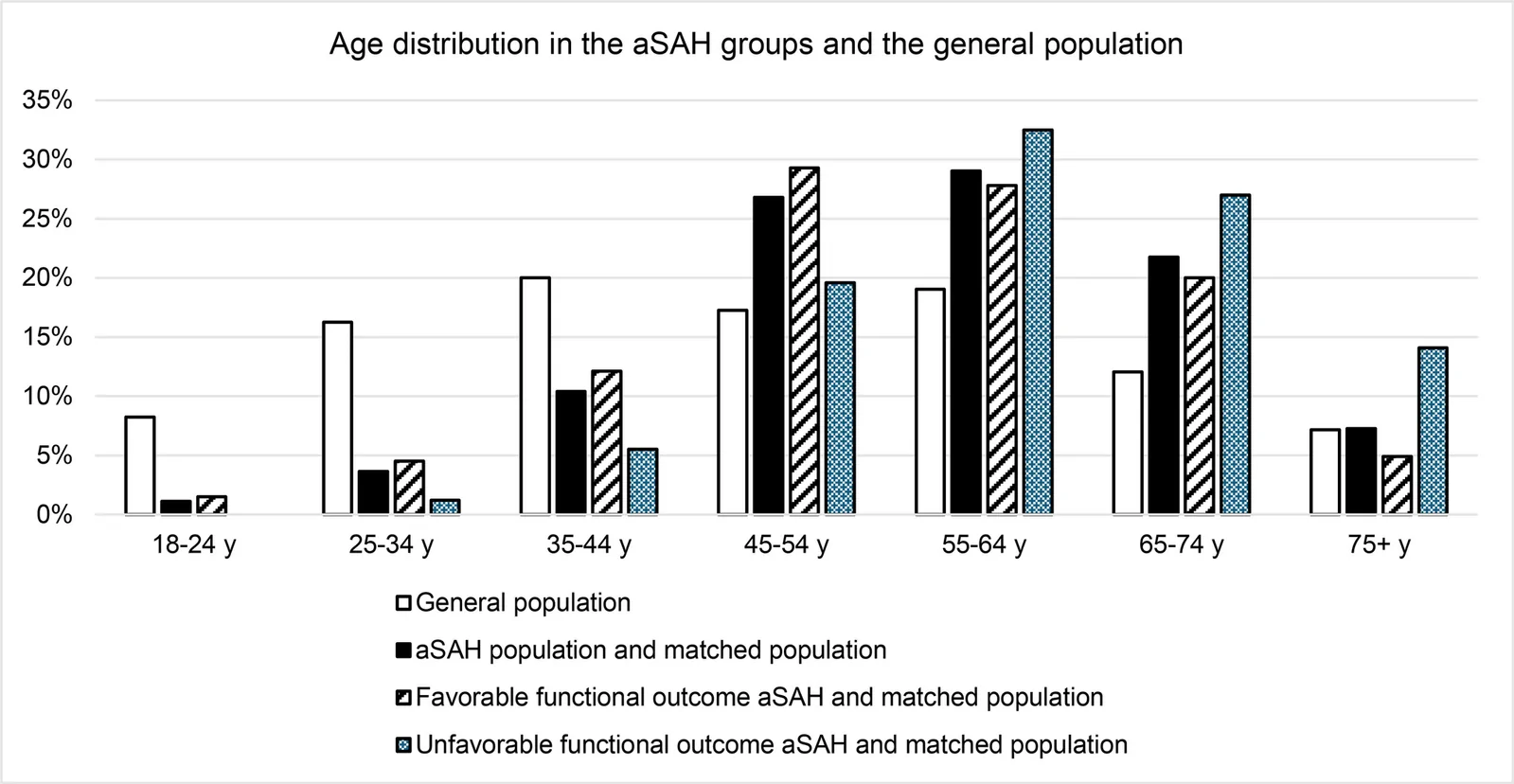
Age distribution among subjects answering the EQ-5D-3L questionnaire in different populations. aSAH = aneurysm subarachnoid hemorrhage. y = years old. General population data as retrieved from published data by Szende et al. [43] unchanged as originally described by Sun et al. in 2012 [42]

Compared with the “Stockholm County general population” used to create the age- and sex-matched reference groups (matched populations) for EQ-5D-3L, all three aSAH populations had an older age distribution (Fig. 2).

### Follow-up data

Follow-up was conducted at a median of 445 days (interquartile range (IQR) 206) after aSAH. Favorable GOSE represented 74.3% (471 patients) of the 634 patients with analyzed data.

**Fig. 3:**
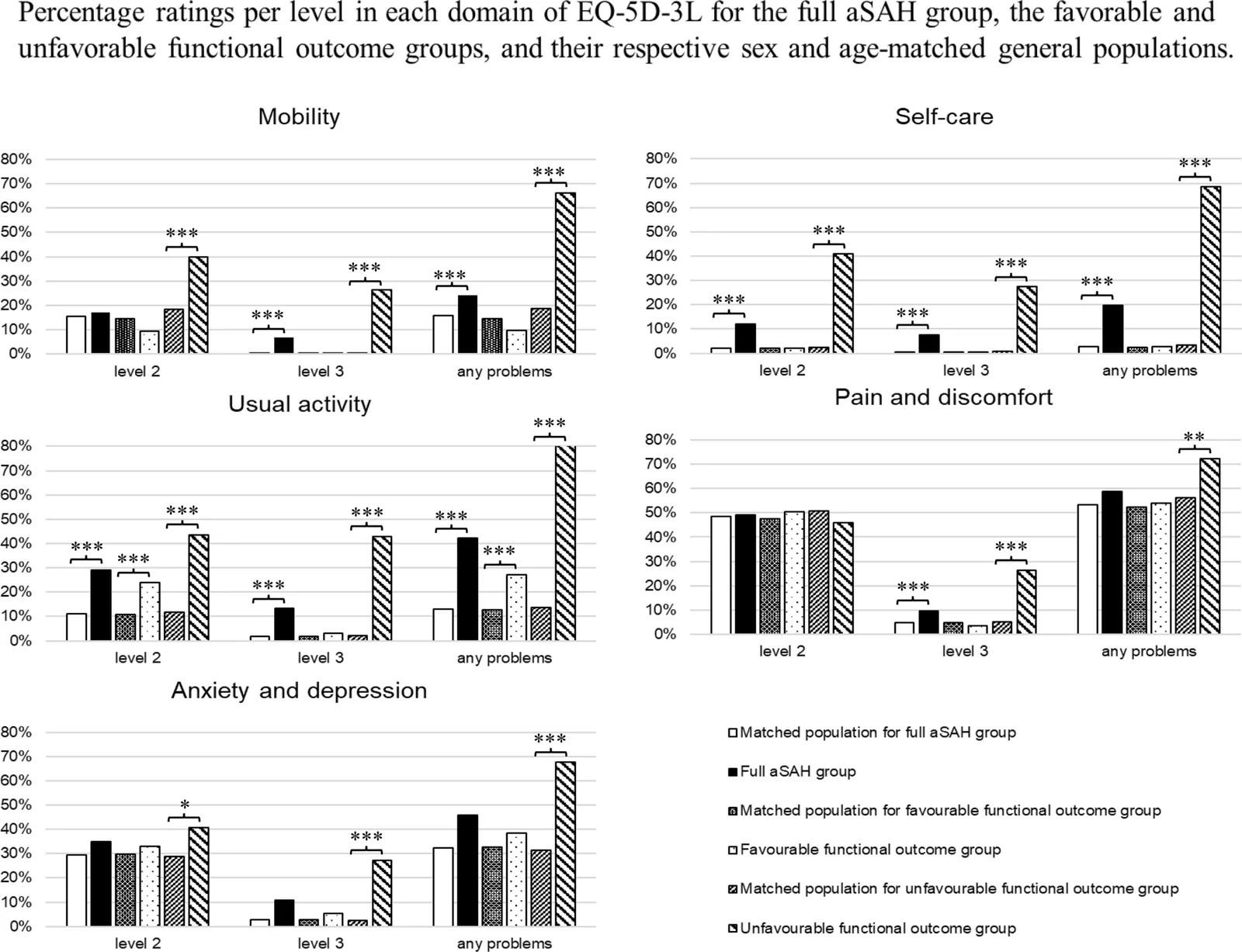
Percentage of ratings per level in each domain of EQ-5D-3L for the aSAH group, the favorable and unfavorable functional outcome groups, and their respective sex- and age-matched general populations. *p ≤ 0.001, **p ≤ 0.0001, ***p ≤ 0.00001. aSAH = aneurysm subarachnoid hemorrhage

The percentages of patients and the corresponding matched population rating level 1, level 2, level 3, or “any problems” in the five domains are presented in Table 2 and Fig. 3.

**Table 2:** Comparison of percentage and number of the sex- and age-matched general population and patients rating a specific level of issues in the favorable functional aSAH group and comparison of odds of rating a specific level of problems in the favorable aSAH group and its matched general population expressed as odds ratios (OR)

		Matched general population	aSAH dGOSE 5–8	OR	*p*
Mobility *n* (%)	**All**	13,018 (100)	471 (100)		
	**L1**	11,109 (85.3)	426 (90.4)		0.002
	**L2**	1,886 (14.5)	44 (9.3)	0.61	0.002
	**L3**	23 (0.2)	1 (0.2)	1.20	0.857
	Any problems*	1,909 (14.7)	45 (9.6)	0.62	0.002
Self-care *n* (%)	**All**	13,017 (100)	471 (100)		
	**L1**	12,685 (97.4)	458 (97.2)		0.777
	**L2**	266 (2.0)	10 (2.1)	1.04	0.905
	**L3**	66 (0.5)	3 (0.6)	1.26	0.698
	Any problems*	332 (2.6)	13 (2.8)	1.09	0.777
Usual activities *n* (%)	**All**	13,015 (100)	471 (100)		
	**L1**	11,383 (87.5)	344 (73.0)		≤ 0.001
	**L2**	1,418 (10.9)	113 (24.0)	2.58	≤ 0.001
	**L3**	214 (1.6)	14 (3.0)	1.83	0.028
	Any problems*	1,632 (12.5)	127 (27.0)	2.58	≤ 0.001
Pain/Discomfort *n* (%)	**All**	13,020 (100)	471 (100)		
	**L1**	6,195 (47.6)	217 (46.1)		0.520
	**L2**	6,199 (47.6)	237 (50.3)	1.11	0.248
	**L3**	626 (4.8)	17 (3.6)	0.74	0.230
	Any problems*	6,825 (52.4)	254 (53.9)	1.06	0.520
Anxiety/Depression *n* (%)	**All**	13,021 (100)	471 (100)		
	**L1**	8,769 (67.3)	290 (61.6)		0.009
	**L2**	3,886 (29.8)	156 (33.1)	1.16	0.127
	**L3**	366 (2.8)	25 (5.3)	1.94	0.002
	Any problems*	4,252 (32.7)	181 (38.4)	1.29	0.009

* Any problems = collapsed level 2 and level 3 issues. *n* number, *d* dichotomized, *aSAH* aneurysm subarachnoid hemorrhage, *GOSE* Glasgow outcome scale extended

ORs to rank level 2, 3 or “any problems” for each respective domain in the groups compared with the matched populations are also presented in Table 2.

Although not significant, the matched population showed 39% higher odds of rating moderate issues (9.3% vs 14.5%; OR 0.61, *p* = 0.002) with mobility than the independent aSAH group and level 3 issues were equal (0.2% vs 0.2%; OR 1.2, *p* = 0.857).

For self-care, the odds of reporting moderate problems for the independent patients were equal to the matched population (2.1% vs 2.0%; OR 1.04, *p* = 0.905) neither was any significant difference reported for extreme issues (0.6% vs 0.5%; OR 1.26, *p* = 0.698).

For usual activities, significant differences from the matched population were found for moderate issues (24.0% vs 10.9%, OR 2.58, *p* ≤ 10^−16) and a trend of higher odds was seen for extreme issues among favorable GOSE patients compared with the matched population (3.0% vs 1.6%; OR 1.83, *p* = 0.030).

No significant difference in pain/discomfort was reported between the independent aSAH group and the matched general population with moderate (50.3% vs 47.6%; OR 1.11, *p* = 0.248) or extreme issues (3.6% vs 4.8%; OR 0.74, *p* = 0.232).

Extreme issues with anxiety/depression were reported almost two times as frequent in the favorable GOSE group compared to the matched general population (5.3% vs 2.8%; OR 1.94, *p* = 0.002). Level 2 issues were reported equally (33.1% vs 29.8%; OR 1.16, *p* = 0.128).

Corresponding data for the entire aSAH group and the unfavorable GOSE 2–4 group are available in Online Resource 1 and Online Resource 3. It shows that the unfavorable GOSE patients were doing worse than the matched population in all levels and dimensions measured. Exception was for moderate problems with pain/discomfort that was reported equally. Instead, 26.4% of the unfavorable GOSE patients reported extreme issues with *P/D* compared to 5.3% of the matched general population (*p* < 0.001).

To illustrate differences in outcome, Online Resource 4, hierarchically orders the reported issues across domains for the independent aSAH group. This ranking contrasts the descriptive percentage of patients reporting issues against the odds ratios (OR) comparing independent aSAH patients to the matched general population. Although the highest percentages of moderate issues were reported in *P/D* (50.3%) and *A/D* (33.1%), the odds ratios indicate that neither rate differs significantly from the matched general population (P/D: OR = 1.11, *p* = 0.248; A/D: OR = 1.16, *p* = 0.128). Extreme issues were most frequently reported (5.3%) in the *A/D* domain which also marked the largest divergence from the matched population (OR 1.94 (*p* = 0.002). Other than this, the ranking order is completely different between the percentage of patients reporting issues in a certain domain and Odds of reporting a certain level of issues in the patient group compared to the matched population.

### VAS results

The global perception of HRQoL in the aSAH group of this study was 68.7 (standard deviation (SD) 19.4). For the unfavorable GOSE group, the mean VAS score was 52.2 (SD 19.7), and for the independent patients, it was 74.1 (SD 16.0).

## Discussion

In this study, we described HRQoL issues after aSAH in relation to functional outcome compared to an age and sex-matched general population. Both the SF-36 [51] and EQ-5D-3L instruments has been used to study HRQoL after aSAH. We have adopted EQ-5D-3L for this national study as it is far less extensive and more easily comprehensible, which is an advantage in regard to patient burden, especially as many might have cognitive issues [3].

Global HRQoL, measured as EQ-5D-VAS is graded lower by patients after aSAH compared with the general population [15, 22, 48, 49]. This study shows a similar global HRQoL (EQ-VAS 68.7) rating a year after aSAH as seen in a previous Swedish study (69.4) [49].

A strong correlation between EQ-VAS score and modified Rankin Scale (mRS) [32, 37] outcome has been shown [14]. mRS and GOSE also provide highly correlated outcomes after aSAH [19]. It is therefore interesting to compare the two studies published before this, where HRQoL (EQ-5D-3L) is assessed among patients with good functional outcome (mRS 0–2) [14, 55] with ours. Custal et al. (2021) highlighted problems with usual activities, anxiety/depression and pain/discomfort as key subjective impairments [14]. Yang et al. (2024) reported a lower EQ-5D index compared to the Korean population, though neither article did compare domain-specific result to a normal population [55]. Yang et al. reported a substantially higher frequency of issues regarding mobility and self-care. Other than that, both studies reported remarkably similar numbers of independent patients with anxiety/depression and issues with usual activities to the present study. However more independent patients reported pain and discomfort in our cohort. Their conclusion was that pain/discomfort and anxiety/depression are domains of concern in the favorable outcome group. While their findings broadly align with ours, their lack of a reference population makes it difficult to definitively attribute these impairments to the aSAH itself. Crucially, we found no statistically significant or clinically relevant difference in pain/discomfort between our independent study group and the matched general population.

### Usual activities

We showed that independent patients rated significantly more issues compared with the matched population in the dimension of usual activities. The results are not surprising, as several other studies present high ratings of issues with usual activities after aSAH [14, 27, 34, 49]. It is encouraging that this is the domain where patients have previously been shown to have the greatest potential of improvement over time [49].

### Anxiety/depression

There was also a clear trend (although not significant) of extreme issues with anxiety/depression among independent patients compared to the matched population in this study. Anxiety/depression is the dimension with second most issues in the general populations in 9 out of 19 countries worldwide [44]. Among older people in Europe, the average reporting of any problems range between 22.9% and 33.9% [8]. Anxiety/depression is also the HRQoL domain with the worst deterioration over time in Sweden, from 33–43% reporting any problems between 1998 and 2002 [10]. Still, many studies suggest anxiety/depression as a major clinical problem following aSAH [14, 35, 48] In one study, 43.3% described any problems, compared with 29.7% in a matched population [48] and two other studies has shown unmatched results of 56.3% [49] and 48.1% [14] respectively.

### Pain/discomfort

Pain/discomfort is the most frequently reported EQ-5D domain in the general population worldwide [44]. For example, across 18 European countries, an average of 36.8% of people aged 50–54 report experiencing pain or discomfort [8]. This prevalence varies by country, reaching 55.2% in Germany compared to 35.9% in Sweden [8]. The frequency of these problems increases steadily within older age groups [8]. We speculate that patients with pain/discomfort issues are allocated to the unfavorable GOSE group, both due to the aSAH, and due to unfavorable baseline characteristics, such as higher frequency of hypertension [23, 25] and higher age [2, 23, 25]. This might explain why patients with favorable functional outcome report less extreme issues with pain and discomfort than the matched population in this study. Partly, it might also be due to limitations with the general population as discussed below.

### Mobility and self-care

The mobility and self-care domains are the two most closely resembling GOSE evaluation criteria and the independent patient group reported equal levels of issues compared to the matched population except for moderate problems with mobility which was reported more often in the matched general population. We conclude that EQ-5D-3L data does not provide additional information to that from GOSE for these two domains regarding independent patients.

Unfavorable GOSE patients reported more problems in all dimensions of EQ5D as compared to their matched general population. This is logical and in line with previous reports [14, 50].

Cognitive issues and fatigue are common after aSAH [39, 52] and self-rating instruments need to be easily comprehensible. EQ-5D-3L fulfils this criterion and can be used for diverse populations and for comparative purposes [43].

### Limitations

Based on an estimated annual incidence of 6–10 spontaneous subarachnoid hemorrhages per 100,000 inhabitants—with 85% attributed to aneurysms—between 1,800 and 3,000 cases were expected to be eligible for inclusion over a 3.5-year period in this study. As noted, there were 1037 aSAH patients with a given consent included. There might be several reasons to the discrepancy. Because we only included patients admitted to a neurosurgical facility, our sample excludes those who died early or were too unstable for transport and subsequent treatment. Unavailability of a study nurse on duty during the stay and the patient's or their relatives' inability to comprehend the study information and make an informed decision at the timepoint probably being the most frequent reasons. The burden of follow up procedures might also have caused refusal to participate, and for a few, language and literacy barriers.

Patients “lost to follow up”, and those not answering the EQ-5D-3L questionnaire might also skew the population studied.

The study group likely has a higher concentration of comorbidities associated with aSAH than the reference group, potentially influencing HRQoL. While age and sex matching should have mitigated the major differences [8, 20, 44, 45], other factors remain. Though rare conditions like genetic predisposition [6], connective tissue disorders [38], and polycystic kidney disease [21] are unlikely to distort aggregate outcomes, they remain potential confounders.

The primary modifiable risk factors—hypertension and smoking [5, 13, 16, 17]—might have direct negative impact on HRQoL [1, 46, 47]. Hypertension affected 34.3% of independent patients and 43.8% of the unfavorable outcome group in this study, compared to 27% of the adult Swedish population [1], and smoking rates were notably higher than Sweden's 11–13% baseline [31], even in the favorable outcome group.

Because this study did not adjust for cigarette smoking or hypertension, negative association with HRQoL may have skewed the results compared to the general population.

As a cross-sectional study, information about changes over time is unavailable. The use of a general population minimizes false interpretations but there is also limitation in the choice of general population data that might skew data. We have used publicly available general population data representing a cross section of Stockholm County in 2006 based on 32,597 survey replies [42, 43]. The great number of participants is an advantage for the indirect standardization, as it makes data stable and less sensitive to chance and outliers. Stockholm County represents over a fifth of the Swedish population [20] and consequently the largest part of the aSAH study group. However, the average age in Stockholm County is younger than in the rest of the country [11], and the incidence of aSAH might be marginally lower. Furthermore, Stockholm County is characterized by a higher average income and educational level compared to the rest of the country [11]. EQ-5D-5L studies has shown that both educational level and average income do impact HRQoL in Sweden [20, 45]. The suspected sociodemographic differences between the matched general population we used and the rest of the country might therefore lead to underestimation of reported issues in our matched general population. Further on, a deterioration of HRQoL over time has been noted in Sweden [10]**.** Therefore, the time-difference in collection of the general population data and the study population might skew data. This is one of the reasons why data from Stockholm County collected in 2006 was chosen as opposed to general population data representative of Sweden as a whole from 1994 with 534 respondents.

## Conclusion

This study provides the first analysis of EQ-5D-3L outcomes in independent patients following aneurysmal subarachnoid hemorrhage (aSAH), compared with an age- and sex-matched general population. Our findings indicate that despite achieving a favorable functional outcome, patients frequently experience difficulties with usual activities as well as anxiety or depression. While previous research has highlighted pain and discomfort as prominent issues in this population, this finding was not confirmed by our data. Furthermore, regarding mobility and self-care, the EQ-5D-3L offers limited incremental insight beyond traditional functional outcome measures (GOSE).

## Supplementary Information

Below is the link to the electronic supplementary material.

Supplementary file1 (PDF 225 KB)

Supplementary file2 (PDF 214 KB)

Supplementary file3 (PDF 181 KB)

Supplementary file4 (PDF 210 KB)

## Data Availability

No datasets were generated or analysed during the current study.

## Abbreviations

aSAH: Aneurysmal subarachnoid hemorrhage
HRQoL: Health related quality of life
EQ-5D-3L: Euro-quality of life—5 dimensions—3 levels
EQ-VAS: EuroQol Visual Analogue Scale
GOSE: Glasgow Outcome Scale Extended
sSAH: Spontaneous SAH
STROBE: Strengthening the reporting of observational studies in epidemiology
BMI: Body mass index
WFNS: World Federation of Neurological Surgeons
CT: Computed tomography
DSA: Digital subtraction angiography
dFisher: Dichotomized Fisher grade
DIND: Delayed ischemic neurological deficit
OR: Odds ratio
