# Supplementary material for: Targeting health-related quality of life issues not found with functional outcome measures a year after aneurysm subarachnoid hemorrhage in a Swedish national cohort
Source: Acta Neurochir (Wien). 2026 Oct 16;168(1):240. doi: 10.1007/s00701-026-07059-z (PMC13676643; doi:10.1007/s00701-026-07059-z)
Supplement: Supplementary file 1 [file 701_2026_7059_MOESM1_ESM.pdf]

Targeting health-related quality of life issues not found with functional outcome measures a year after aneurysm subarachnoid hemorrhage in a Swedish national cohort

ACTA Neurochirurgica

Helena Aineskog MD[1*], Bryndís Baldvinsdóttir MD PhD[2], Ola G. Nilsson MD PhD[2], Asgeir S. Jakola MD PhD[3], Erik Kronvall MD PhD[2], Elisabeth Ronne Engström MD PhD[4], Paula Klurfan MD[3], Peter Alpkvist MD[5], Mikael Svensson MD PhD[5], Jan Hillman MD PhD[6], Peter Lindvall MD PhD[1]

[1] Department of Clinical Sciences - Neurosciences, Umeå University, Umeå, Sweden

[2] Department of Clinical Sciences - Neurosurgery, Lund University, Lund, Sweden

[3] Institute of Neuroscience and Physiology, Department of Clinical Neuroscience, Sahlgrenska Academy, University of Gothenburg, Gothenburg, Sweden

[4] Department of Medical Sciences, Section of Neurosurgery, Uppsala University, Uppsala, Sweden

[5] Department of Clinical Neuroscience, Karolinska Institute, Stockholm, Sweden

[6] Department of Clinical Sciences, Linköping University, Linköping, Sweden

Correspondence:

Helena.Aineskog@umu.se

Online Resource 1 *Descriptive characteristics.*

		Total*	Full cohort		Total*	Unfavorable GOSE	
		n	**n=634**		n	**n=163**	
**Age** (yrs) median (min–max), IQR		634	57 (19–88)	17	163	61 (28–88)	16
**18–24** (yrs) n, %		634	7	1.1%	163	0	0.0%
**25–34** (yrs) n, %		634	23	3.6%	163	2	1.2%
**35–44** (yrs) n, %		634	66	10.4%	163	9	5.5%
**45–54** (yrs) n, %		634	170	26.8%	163	32	19.6%
**55–64** (yrs) n, %		634	184	29.0%	163	53	32.5%
**65–74** (yrs) n, %		634	138	21.8%	163	44	27.0%
**75+** (yrs) n, %		634	46	7.3%	163	23	14.1%
**Sex** (female) n, %		634	452	71.3%	163	110	67.5%
**Current smoking,** n, %		607	218	35.9%	152	69	45.4%
**BMI** (kg/m^2) mean (min–max), SD		586	26.5 (16–49)	5.28	148	27.0 (16–47)	5.8
**Hypertension**		632	232	36.7%	162	71	43.8%
**Diabetes**		633	20	3.2%	163	10	6.1%
**Previous stroke**		633	22	3.5%	163	10	6.1%
**Psychiatric diagnosis**		633	54	8.5%	163	18	11.0%
**Kidney disease**		633	9	1.4%	163	4	2.5%
**dWFNS (poor=3–5)**		561	173	30.8%	147	83	50.9%
**WFNS grade**							
	**1**		234	41.7%		28	19.0%
	**2**		154	27.5%		36	24.5%
	**3**		18	3.2%		10	6.8%
	**4**		121	21.6%		53	36.1%
	**5**		34	6.1%		20	13.6%
**dFisher (4)**		634	270	42.6%	163	97	59.5%
**Hemiparesis**		551	57	10.3%	141	37	26.2%
**Location**					163		
	**ICA+**	173	27.3%	27.3%		33	20.2%
	**MCA**	135	21.3%	21.3%		44	27.0%
	**Anterior complex**	255	40.2%	40.2%		68	41.7%
	**Posterior circulation**	71	11.2%	11.2%		18	11.0%
**Treatment**					163		
	**Microsurgical**	201	31.7%	31.7%		51	31.3%
	**Endovascular**	429	67.7%	67.7%		110	67.5%
	**Both**	4	0.6%	0.6%		2	1.2%
**Meningitis**		630	54	8.6%	162	27	16.7%
**DIND**		620	136	21.9%	155	53	34.2%
**Ictus to treatment** (h) median (IQR)		595	77	146	150	68	129
**Hospital stay** (d) median (IQR)		613	15	10	161	17	10
**Admission to follow-up** (d) median (IQR)		605	432	169	159	407	146
**Seizure**		627	38	6.1%	159	19	11.9%
**Shunt dependency**		619	130	21.0%	158	67	42.4%

Footnote, Online Resource 1 *Descriptive characteristics of the aSAH populations (full group and unfavorable GOSE (2–4). *Number analyzed in the group. IQR=interquartile range, yrs=years, n=number, BMI=body mass index, SD=standard deviation, d=dichotomized, ICA+=internal carotid artery and posterior communicating artery, MCA=middle cerebral artery. aSAH=aneurysm subarachnoid hemorrhage, dFisher=dichotomized Fisher grade, WFNS=world federation of Neurosurgical Societies functional outcome scale, DIND=delayed ischemic neurologic deficit, GOSE=Glasgow outcome scale extended*
