# Supplementary material for: Targeting health-related quality of life issues not found with functional outcome measures a year after aneurysm subarachnoid hemorrhage in a Swedish national cohort
Source: Acta Neurochir (Wien). 2026 Oct 16;168(1):240. doi: 10.1007/s00701-026-07059-z (PMC13676643; doi:10.1007/s00701-026-07059-z)
Supplement: Supplementary file 2 [file 701_2026_7059_MOESM2_ESM.pdf]

Targeting health-related quality of life issues not found with functional outcome measures a year after aneurysm subarachnoid hemorrhage in a Swedish national cohort

ACTA Neurochirurgica

Helena Aineskog MD[1*], Bryndís Baldvinsdóttir MD PhD[2], Ola G. Nilsson MD PhD[2], Asgeir S. Jakola MD PhD[3], Erik Kronvall MD PhD[2], Elisabeth Ronne Engström MD PhD[4], Paula Klurfan MD[3], Peter Alpkvist MD[5], Mikael Svensson MD PhD[5], Jan Hillman MD PhD[6], Peter Lindvall MD PhD[1]

[1] Department of Clinical Sciences - Neurosciences, Umeå University, Umeå, Sweden

[2] Department of Clinical Sciences - Neurosurgery, Lund University, Lund, Sweden

[3] Institute of Neuroscience and Physiology, Department of Clinical Neuroscience, Sahlgrenska Academy, University of Gothenburg, Gothenburg, Sweden

[4] Department of Medical Sciences, Section of Neurosurgery, Uppsala University, Uppsala, Sweden

[5] Department of Clinical Neuroscience, Karolinska Institute, Stockholm, Sweden

[6] Department of Clinical Sciences, Linköping University, Linköping, Sweden

Correspondence:

Helena.Aineskog@umu.se

Online Resource 2 *Comparison of percentage and number of responders and patients rating a specific level of issues in the full aSAH group and the unfavorable functional outcome group and their sex- and age-matched general population groups and comparison of odds of rating a specific level of issues for each EQ-dimension.*

		Matched full population	aSAH full population n=634	OR	p	Matched dGOSE 2–4 population	aSAH dGOSE 2–4 n=163	OR	p
**Mobility** n (%)	**All**	13,121 (100)	634 (100)			11,570 (100)	163 (100)		
	**L1**	11061 (84.3)	481 (75.9)		≤0.001	9,406 (81.3)	55 (33.7)		≤0.001
	**L2**	2,035 (15.5)	109 (17.2)	1.13	0.254	2,135 (18.5)	65 (39.9)	2.93	≤0.001
	**L3**	25 (0.2)	44 (6.9)	39.07	≤0.001	29 (0.3)	43 (26.4)	142.6	≤0.001
	**Any problems***	2,060 (15.7)	153 (24.1)	1.71	≤0.001	2,164 (18.7)	108 (66.3)	8.54	≤≤0.001
**Self-care** n (%)	**All**	13,121 (100)	634 (100)			11,573 (100)	163 (100)		
	**L1**	12761 (97.3)	509 (80.3)		≤0.001	11,196 (96.7)	51 (31.3)		≤0.001
	**L2**	287 (2.2)	77 (12.1)	6.18	≤0.001	294 (2.5)	67 (41.1)	26.72	≤0.001
	**L3**	73 (0.6)	48 (7.6)	14.64	≤0.001	83 (0.7)	45 (27.6)	52.79	≤0.001
	**Any problems***	360 (2.7)	125 (19.7)	8.71	≤0.001	377 (3.3)	112 (68.7)	65.21	≤0.001
**Usual activities** n (%)	**All**	13,122 (100)	634 (100)			11,574 (100)	163 (100)		
	**L1**	11,434 (87.1)	366 (57.7)		≤0.001	9,977 (86.2)	22 (13.5)		≤0.001
	**L2**	1,457 (11.1)	184 (29.0)	3.27	≤0.001	1,355 (11.7)	71 (43.6)	5.82	≤0.001
	**L3**	231 (1.8)	84 (13.2)	8.52	≤0.001	242 (2.1)	70 (42.9)	35.25	≤0.001
	**Any problems***	1,688 (12.9)	268 (42.3)	4.96	≤0.001	1,597 (13.8)	141 (86.5)	40.04	≤0.001
**Pain/Discomfort** n (%)	**All**	13,124 (100)	634 (100)			11,572 (100)	163 (100)		
	**L1**	6,118 (46.6)	262 (41.3)		0.009	5,073 (43.8)	45 (27.6)		≤0.001
	**L2**	6,360 (48.5)	312 (49.2)	1.03	0.712	5,890 (50.9)	75 (46.0)	0.82	0.215
	**L3**	646 (4.9)	60 (9.5)	2.02	≤0.001	609 (5.3)	43 (26.4)	6.45	≤0.001
	**Any problems***	7,006 (53.4)	372 (58.7)	1.24	0.009	6,499 (56.2)	118 (72.4)	2.05	≤0.001
**Anxiety/Depression** n (%)	**All**	13,122 (100)	633 (100)			11,571 (100)	162 (100)		
	**L1**	8,883 (67.7)	342 (54.0)		≤0.001	7,951 (68.7)	52 (32.1)		≤0.001
	**L2**	3,877 (29.5)	222 (35.1)	1.29	0.003	3,321 (28.7)	66 (40.7)	1.71	0.001
	**L3**	362 (2.8)	69 (10.9)	4.31	≤0.001	299 (2.6)	44 (27.2)	14.06	≤0.001
	**Any problems***	4,239 (32.3)	291 (46.0)	1.78	≤0.001	3,620 (31.3)	110 (67.9)	4.65	≤0.001

Footnote, Online Resource 2 *Odds of the full aSAH group and the unfavorable GOSE group rating a specific level of problems for each domain compared with the odds of the two matched populations rating the same level of problems, expressed as odds ratios (ORs).* Any problems = collapsed level 2 and level 3 issues. n=number, d=dichotomized. aSAH=aneurysm subarachnoid hemorrhage, GOSE=Glasgow outcome scale extended*
