# Supplementary material for: Targeting health-related quality of life issues not found with functional outcome measures a year after aneurysm subarachnoid hemorrhage in a Swedish national cohort
Source: Acta Neurochir (Wien). 2026 Oct 16;168(1):240. doi: 10.1007/s00701-026-07059-z (PMC13676643; doi:10.1007/s00701-026-07059-z)
Supplement: Supplementary file 3 [file 701_2026_7059_MOESM3_ESM.pdf]

Targeting health-related quality of life issues not found with functional outcome measures a year after aneurysm subarachnoid hemorrhage in a Swedish national cohort

ACTA Neurochirurgica

Helena Aineskog MD[1*], Bryndís Baldvinsdóttir MD PhD[2], Ola G. Nilsson MD PhD[2], Asgeir S. Jakola MD PhD[3], Erik Kronvall MD PhD[2], Elisabeth Ronne Engström MD PhD[4], Paula Klurfan MD[3], Peter Alpkvist MD[5], Mikael Svensson MD PhD[5], Jan Hillman MD PhD[6], Peter Lindvall MD PhD[1]

[1] Department of Clinical Sciences - Neurosciences, Umeå University, Umeå, Sweden

[2] Department of Clinical Sciences - Neurosurgery, Lund University, Lund, Sweden

[3] Institute of Neuroscience and Physiology, Department of Clinical Neuroscience, Sahlgrenska Academy, University of Gothenburg, Gothenburg, Sweden

[4] Department of Medical Sciences, Section of Neurosurgery, Uppsala University, Uppsala, Sweden

[5] Department of Clinical Neuroscience, Karolinska Institute, Stockholm, Sweden

[6] Department of Clinical Sciences, Linköping University, Linköping, Sweden

Correspondence:

Helena.Aineskog@umu.se

Online Resource 3 Comparison of odds of rating a specific level of issues for each EQ-dimension.

**All**	**Level 2**		**Level 3**		**Any problems**	
	OR	p	OR	p	OR	p
**Mobility**	1.13	0.254	39.07	≤0.001	1.71	≤0.001
**Self-care**	6.18	≤0.001	14.64	≤0.001	8.71	≤0.001
**Usual activities**	3.27	≤0.001	8.52	≤0.001	4.96	≤0.001
**Pain/Discomfort**	1.03	0.712	2.02	≤0.001	1.24	0.009
**Anxiety/Depression**	1.29	0.003	4.31	≤0.001	1.78	≤0.001
**Unfavorable GOSE**	**Level 2**		**Level 3**		**Any problems**	
	OR	p	OR	p	OR	p
**Mobility**	2.93	≤0.001	142.60	≤0.001	8.54	≤0.001
**Self-care**	26.78	≤0.001	52.79	≤0.001	65.21	≤0.001
**Usual activities**	5.82	≤0.001	35.25	≤0.001	40.04	≤0.001
**Pain/Discomfort**	0.82	0.216	6.45	≤0.001	2.05	≤0.001
**Anxiety/Depression**	1.71	0.001	14.06	≤0.001	4.65	≤0.001

Online Resource 3 *Odds of the full aSAH group and the unfavorable GOSE group rating a specific level of problems for each domain compared with the odds of the two matched populations rating the same level of problems, expressed as odds ratios (ORs). aSAH aneurysm subarachnoid hemorrhage, GOSE=Glasgow outcome scale extended, EQ=Euro Quality of life*
