# Supplementary material for: Targeting health-related quality of life issues not found with functional outcome measures a year after aneurysm subarachnoid hemorrhage in a Swedish national cohort
Source: Acta Neurochir (Wien). 2026 Oct 16;168(1):240. doi: 10.1007/s00701-026-07059-z (PMC13676643; doi:10.1007/s00701-026-07059-z)
Supplement: Supplementary file 4 [file 701_2026_7059_MOESM4_ESM.pdf]

Targeting health-related quality of life issues not found with functional outcome measures a year after aneurysm subarachnoid hemorrhage in a Swedish national cohort

ACTA Neurochirurgica

Helena Aineskog MD[1*], Bryndís Baldvinsdóttir MD PhD[2], Ola G. Nilsson MD PhD[2], Asgeir S. Jakola MD PhD[3], Erik Kronvall MD PhD[2], Elisabeth Ronne Engström MD PhD[4], Paula Klurfan MD[3], Peter Alpkvist MD[5], Mikael Svensson MD PhD[5], Jan Hillman MD PhD[6], Peter Lindvall MD PhD[1]

[1] Department of Clinical Sciences - Neurosciences, Umeå University, Umeå, Sweden

[2] Department of Clinical Sciences - Neurosurgery, Lund University, Lund, Sweden

[3] Institute of Neuroscience and Physiology, Department of Clinical Neuroscience, Sahlgrenska Academy, University of Gothenburg, Gothenburg, Sweden

[4] Department of Medical Sciences, Section of Neurosurgery, Uppsala University, Uppsala, Sweden

[5] Department of Clinical Neuroscience, Karolinska Institute, Stockholm, Sweden

[6] Department of Clinical Sciences, Linköping University, Linköping, Sweden

Correspondence:

Helena.Aineskog@umu.se

Online Resource 4 *Comparison of the ranking order of issues within each domain, based on whether outcomes are described as Odds Ratios or as percentages reporting issues in the target group, without comparison to a matched general population.*

Level 2									
RANKING ORDER	**Favorable**			**Unfavorable**			**All**		
	%	OR	DISCR*	%	OR	DISCR*	%	OR	DISCR*
**1**	Pain/Discomfort<br>%: 50.3<br>n: 237	Usual activities<br>OR: 2.58<br>p≤0.001	**YES**	Pain/Discomfort<br>%: 46.0<br>n: 75	Self-care<br>OR: 26.78<br>p≤0.001	**YES**	Pain/Discomfort<br>%: 49.2<br>n: 312	Self-care<br>OR: 6.18<br>p≤0.001	**YES**
**2**	Anxiety/Depression<br>%: 33.1<br>n: 156	Anxiety/Depression<br>OR: 1.16<br>p=0.128	**NO**	Usual activities<br>%: 43.6<br>n: 71	Usual activities<br>OR: 5.82<br>p≤0.001	**NO**	Anxiety/Depression<br>%: 35.1<br>n: 222	Usual activities<br>OR: 3.27<br>p≤0.001	**YES**
**3**	Usual activities<br>%: 24.0<br>n: 113	Pain/Discomfort<br>OR: 1.11<br>p=0.248	**YES**	Self-care<br>%: 41.1<br>n: 67	Mobility<br>OR: 2.93<br>p≤0.001	**YES**	Usual activities<br>%: 29.0<br>n: 184	Anxiety/Depression<br>OR: 1.29<br>P=0.003	**YES**
**4**	Mobility<br>%: 9.3<br>n: 44	Self-care<br>OR: 1.04<br>p=0.905	**YES**	Anxiety/Depression<br>%: 40.7<br>n: 66	Anxiety/Depression<br>OR: 1.71<br>p=0.001	**NO**	Mobility<br>%: 17.2<br>n: 109	Mobility<br>OR: 1.13<br>p=0.254	**NO**
**5**	Self-care<br>%: 2.1<br>n: 10	Mobility<br>OR: 0.61<br>p=0.002	**YES**	Mobility<br>%: 39.9<br>n: 65	Pain/Discomfort<br>OR: 0.82<br>p=0.216	**YES**	Self-care<br>%: 12.1<br>n: 77	Pain/Discomfort<br>OR: 1.03<br>P=0.712	**YES**

Level 3									
RANKING ORDER	**Favorable**			**Unfavorable**			**All**		
	%	OR	DISCR*	%	OR	DISCR*	%	OR	DISCR*
**1**	Anxiety/Depression<br>%: 5.3<br>n: 25	Anxiety/Depression<br>OR: 1.94<br>P=0.002	**NO**	Usual activities<br>%: 42.9<br>n: 70	Mobility<br>OR: 142.60<br>p≤0.001	**YES**	Usual activities<br>%: 13.2<br>n: 84	Mobility<br>OR: 39.07<br>p≤0.001	**YES**
**2**	Pain/Discomfort<br>%: 3.6<br>n: 17	Usual activities<br>OR: 1.83<br>p=0.030	**YES**	Self-care<br>%: 27.6<br>n: 45	Self-care<br>OR: 52.79<br>p≤0.001	**NO**	Anxiety/Depression<br>%: 10.9<br>n: 69	Self-care<br>OR: 14.64<br>p≤0.001	**YES**
**3**	Usual activities<br>%: 3.0<br>n: 14	Self-care<br>OR: 1.26<br>P=0.698	**YES**	Anxiety/Depression<br>%: 27.2<br>n: 44	Usual activities<br>OR: 35.25<br>p≤0.001	**YES**	Pain/Discomfort<br>%: 9.5<br>n: 60	Usual activities<br>OR: 8.52<br>p≤0.001	**YES**
**4**	Self-care<br>%: 0.6<br>n: 3	Mobility<br>OR: 1.20<br>p=0.857	**YES**	Mobility<br>%: 26.4<br>n: 43	Anxiety/Depression<br>OR: 14.06<br>p≤0.001	**YES**	Self-care<br>%: 7.6<br>n: 48	Anxiety/Depression<br>OR: 4.31<br>p≤0.001	**YES**
**5**	Mobility<br>%: 0.2<br>n: 1	Pain/Discomfort<br>OR: 0.74<br>p=0.232	**YES**	Pain/Discomfort<br>%: 26.4<br>n: 43	Pain/Discomfort<br>OR: 6.45<br>p≤0.001	**NO**	Mobility<br>%: 6.9<br>n: 44	Pain/Discomfort<br>OR: 2.02<br>p≤0.001	**YES**

Any problems									
RANKING ORDER	**Favorable**			**Unfavorable**			**All**		
	%	OR	DISCR*	%	OR	DISCR*	%	OR	DISCR*
**1**	Pain/Discomfort<br>%: 53.9<br>n: 254	Usual activities<br>OR: 2.58<br>p≤0.001	**YES**	Usual activities<br>%: 86.5<br>n: 141	Self-care<br>OR: 65.21<br>p≤0.001	**YES**	Pain/Discomfort<br>%: 58.7<br>n: 372	Self-care<br>OR: 8.71<br>p≤0.001	**YES**
**2**	Anxiety/Depression<br>%: 38.4<br>n: 181	Anxiety/Depression<br>OR: 1.29<br>P=0.009	**NO**	Pain/Discomfort<br>%: 72.4<br>n: 118	Usual activities<br>OR: 40.04<br>p≤0.001	**YES**	Anxiety/Depression<br>%: 46.0<br>n: 291	Usual activities<br>OR: 4.96<br>p≤0.001	**YES**
**3**	Usual activities<br>%: 27.0<br>n: 127	Self-care<br>OR: 1.09<br>P=0.777	**YES**	Self-care<br>%: 68.7<br>n: 112	Mobility<br>OR: 8.54<br>p≤0.001	**YES**	Usual activities<br>%: 42.3<br>n: 268	Anxiety/Depression<br>OR: 1.78<br>p≤0.001	**YES**
**4**	Mobility<br>%: 9.6<br>n: 45	Pain/Discomfort<br>OR: 1.06<br>P=0.520	**YES**	Anxiety/Depression<br>%: 67.9<br>n: 110	Anxiety/Depression<br>OR: 4.65<br>p≤0.001	**NO**	Mobility<br>%: 24.1<br>n: 153	Mobility<br>OR: 1.71<br>p≤0.001	**NO**
**5**	Self-care<br>%: 2.8<br>n: 13	Mobility<br>OR: 0.62<br>p=0.002	**YES**	Mobility<br>%: 66.3<br>n: 108	Pain/Discomfort<br>OR: 2.05<br>p≤0.001	**YES**	Self-care<br>%: 19.7<br>n: 125	Pain/Discomfort<br>OR: 1.24<br>P=0.009	**YES**

Online Resource 4 *The ranking order of what domain had most ratings at a specific level (2, 3, or any problems) for aneurysm subarachnoid hemorrhage (aSAH) patients with favorable outcome, unfavorable outcome, and all. % = The ranking order is determined based on the percentage of patients rating a specific level in each group. OR = The ranking order is determined based on the odds of the aSAH group (favorable, unfavorable, or all) rating the specific level of issues compared with that of its matched population doing the same. *DISCR = discrepancy between the ranking order of a certain domain depending on the way of describing outcome*
